# Benchmarking next- versus third-generation sequencing in metagenomics: performance metrics and diagnostic efficacy

**DOI:** 10.1128/spectrum.03993-25

**Published:** 2026-06-03

**Authors:** Jian Hu, Hua Zhang, Hui Miao, Wenjing Chang, Jianpo Zheng, Fangfang Hu, Dongdan Zhang, Weiqing Guo, Peng Hu, Rui Han, Jing Wang, Lifeng Li, Xiaoqin Wang

**Affiliations:** 1 Department of Clinical Laboratory, The First Affiliated Hospital of Xi’an Jiaotong University, Shaanxi, China; 2 Department of Laboratory Medicine, Guizhou Provincial People’ s Hospital, Guizhou, China; 3 Genskey Medical Technology Co., Ltd, Beijing, China; Central Texas Veterans Health Care System, Temple, Texas, USA

**Keywords:** metagenomic sequencing, next-generation sequencing, host-depleted nanopore sequencing, bronchoalveolar lavage fluid, polymicrobial infection

## Abstract

This study aimed to compare the analytical characteristics and diagnostic performance of short-read next-generation sequencing (NGS) and long-read third-generation sequencing (TGS) for metagenomic pathogen detection, using defined mock communities and clinical bronchoalveolar lavage fluid (BALF) samples. Mock evaluations included microbe-host gradient mixtures (D1/D2) and six complex microbial panels (M1–M6). Sequencing was performed on Illumina, MGI, and Oxford Nanopore Technologies (ONT) platforms. Clinical validation was conducted on 62 BALF samples. Diagnostic performance was assessed against culture, clinical microbiological tests (CMT), and a composite reference standard (CRS). Turnaround times for Illumina and MGI were approximately 18–20 h and 14–19 h, respectively, whereas the ONT workflow was completed within 4–6 h. The microbe-to-host DNA ratio significantly influenced sequencing performance. Depletion of host DNA notably enhanced ONT detection, reducing the false-negative rate for low-abundance microorganisms from 43.3% to 6.7%. For all mock samples, both the Illumina and MGI platforms demonstrated 100% sensitivity and showed highly concordant detection profiles. In clinical specimens, when evaluated against the composite reference standard, the positive percent agreement (PPA) values of NGS and TGS were 93.3% and 90.7%, respectively, with corresponding negative percent agreements (NPAs) of 77.6% and 83.3%. Both platforms identified numerous pathogens that were missed by culture, especially in polymicrobial infections. Among 22 CRS-defined polymicrobial samples, culture identified all pathogens in only 2 cases, whereas NGS and TGS achieved full pathogen recovery in 18 and 17 cases, respectively. Within the evaluated workflows, short-read sequencing showed slightly higher sensitivity and overall stability, whereas host-depleted ONT offered a substantial turnaround-time advantage and may serve as a useful complementary approach in complex or time-sensitive clinical scenarios.

**IMPORTANCE:**

Rapid and accurate identification of the microbes causing pneumonia is essential for choosing effective treatment, yet current diagnostic tests are slow and often miss important pathogens. We systematically compared two major DNA sequencing strategies—established short-read platforms and newer long-read nanopore sequencing—using both carefully designed mock communities and real bronchoalveolar lavage samples from patients. We show when removal of human DNA is essential, how mixed infections are best captured, and what trade-offs exist between speed and sensitivity. Our results provide practical guidance on how hospitals can implement sequencing-based diagnostics, when rapid nanopore testing can complement conventional short-read workflows, and how to interpret sequencing read counts in day-to-day clinical decision-making.

## INTRODUCTION

Metagenomic sequencing has revolutionized infectious disease diagnostics by enabling comprehensive, hypothesis-free detection of pathogens directly from clinical or environmental samples (1). Unlike traditional microbiological approaches, which rely on cultivation or targeted amplification, metagenomic workflows simultaneously identify bacteria, fungi, viruses, and parasites within a single assay, allowing rapid and accurate characterization of complex microbial communities (2, 3). As sequencing costs decline and analytical pipelines mature, metagenomics has increasingly become a central component of clinical microbiology, outbreak tracing, and antimicrobial resistance surveillance (4, 5).

Short-read next-generation sequencing (NGS), typified by Illumina and MGI platforms, has long served as the cornerstone of metagenomic applications due to its high base-calling accuracy, scalability, and well-established bioinformatics infrastructure (6, 7). These platforms generate millions of reads with low error rates, facilitating robust pathogen identification and resistance gene profiling (8). However, short reads are suboptimal for resolving repetitive regions, reconstructing complete genomes, or discriminating closely related strains, and the need for batched library preparation and sequencing can prolong turnaround time (9), which may limit their utility for urgent clinical decision-making.

The advent of long-read third-generation sequencing (TGS) technologies, such as Oxford Nanopore Technologies (ONT) and PacBio, has introduced new opportunities for real-time, high-contiguity pathogen characterization (10, 11). ONT in particular enables continuous data acquisition and on-the-fly analysis, allowing same-day pathogen detection and structural variant assessment directly from clinical specimens (12–14). Long reads also facilitate more complete genome assembly and improved resolution of mobile genetic elements. Nonetheless, long-read platforms still face important challenges in clinical metagenomics, including higher per-base error rates compared with short-read NGS and limited sequencing throughput (15). In addition, the predominance of host DNA in many specimen types can dilute microbial signals, reducing sensitivity for low-abundance pathogens unless effective strategies are implemented.

These considerations underscore the need for further evaluation of short- and long-read sequencing in clinically relevant settings. In this study, we conducted a comparative evaluation of two widely implemented short-read metagenomic workflows (Illumina NextSeq 550 and MGISEQ-200) and one long-read workflow (Oxford Nanopore GridION) using both defined mock communities and clinical samples. Illumina NextSeq 550 and MGISEQ-200 were included as representative short-read platforms to assess the consistency and analytical performance of short-read workflows, whereas ONT was included as a representative long-read platform because of its real-time sequencing capability and potential value in time-sensitive clinical settings.

## MATERIALS AND METHODS

### Study design

**Fig 1:**
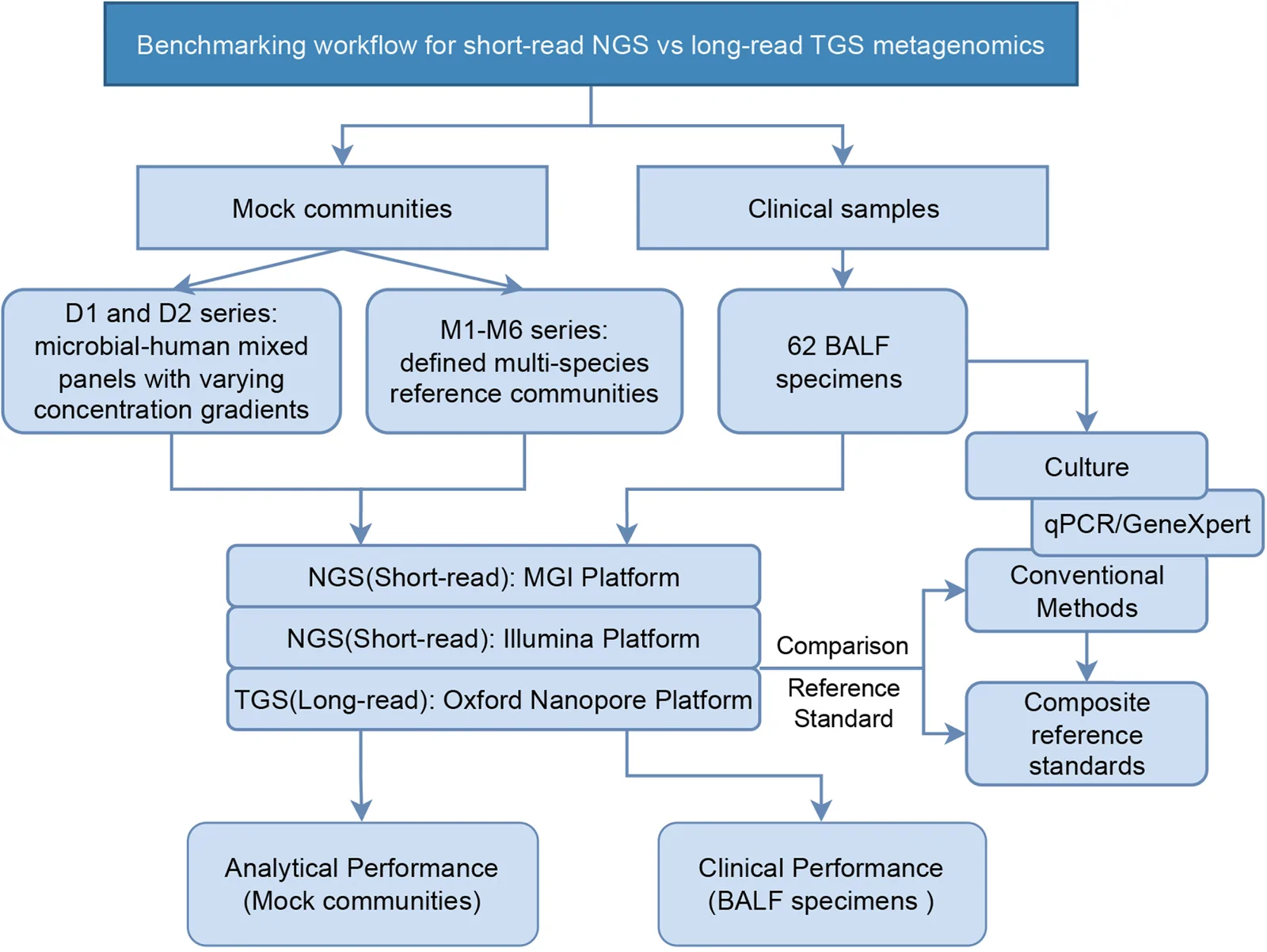
Flowchart of this study. BALF, bronchoalveolar lavage fluid; PPA, positive percent agreement; NPA, negative percent agreement.

To compare clinically relevant short-read and long-read metagenomic sequencing workflows, we analyzed both defined mock communities and clinical bronchoalveolar lavage fluid (BALF) specimens (Fig. 1). First, defined mock panels were used to characterize analytical performance under controlled conditions, including the impact of host background and multi-organism co-detection. Second, BALF specimens were used to assess clinical performance and value against conventional microbiological testing and a composite reference standard.

### Mock communities

Two mock panels were used: the D1/D2 microbe-host gradient series and the M-series defined multi-species reference panels.

### D1/D2 reference panels

The D1 and D2 reference panels were provided by the National Institutes for Food and Drug Control and were constructed as standardized microbe-host gradient mixtures. D1 comprised five bacterial strains from the American Type Culture Collection (ATCC): *Haemophilus parainfluenzae* (ATCC 9796), *Acinetobacter junii* (ATCC 17908), *Listeria grayi* (ATCC 700545), *Rhodococcus hoagii* (ATCC 6939), and *Micrococcus luteus* (ATCC 49732). D2 comprised four bacteria and one fungus: *Legionella pneumophila* (ATCC 33152), *Pseudomonas fluorescens* (ATCC 13525), *Neisseria sicca* (ATCC 9913), *Aeromonas hydrophila* (ATCC 7966), and *Clavispora lusitaniae* (ATCC 34449).

Each series consisted of five microbe-to-host conditions (human cells:microorganisms): 10^5^:10^5^ (D1-1/D2-1), 10^5^:10^4^ (D1-2/D2-2), 10^5^:10^3^ (D1-3/D2-3), 10^4^:10^3^ (D1-4/D2-4), and 10^3^:10^3^ (D1-5/D2-5) (Table S1).

### M-series reference panels

The M-series mock panels consisted of 29 clinically relevant ATCC strains, including 12 Gram-negative bacteria, 11 Gram-positive bacteria, and 6 fungi, spanning genome sizes of 1.8–14.7 Mb and GC contents of 32.7%–73.0% (Table S2). The 29 strains were *Acinetobacter baumannii* (ATCC 9955), *Acinetobacter junii* (ATCC 17908), *Aeromonas hydrophila* (ATCC 7966), *Candida albicans* (ATCC 10231), *Candida glabrata* (ATCC 15545), *Candida parapsilosis* (ATCC 90875), *Candida tropicalis* (ATCC 66029), *Clavispora lusitaniae* (ATCC 34449), *Enterobacter cloacae* (ATCC 13047), *Enterococcus faecalis* (ATCC 700802), *Enterococcus faecium* (ATCC 700221), *Escherichia coli* (ATCC 43888), *Haemophilus influenzae* (ATCC 10211), *Klebsiella oxytoca* (ATCC 13182), *Klebsiella pneumoniae* (ATCC BAA-1705), *Listeria grayi* (ATCC 700545), *Listeria monocytogenes* (ATCC 19115), *Micrococcus luteus* (ATCC 49732), *Neisseria meningitidis* (ATCC 43744), *Pichia kudriavzevii* (ATCC 90878), *Proteus mirabilis* (ATCC 29906), *Pseudomonas aeruginosa* (ATCC 27853), *Rhodococcus hoagii* (ATCC 6939), *Serratia marcescens* (ATCC 27137), *Staphylococcus aureus* (ATCC BAA-1747), *Staphylococcus epidermidis* (ATCC 12228), *Streptococcus mitis* (ATCC 15914), *Streptococcus agalactiae* (ATCC 13813), and *Streptococcus pyogenes* (ATCC 19615).

To generate multiple positive-control panels with manageable complexity and mixed microbial types, these strains were organized into six reference communities (M1–M6), with each community containing at least two microbial categories (Gram-positive, Gram-negative, and/or fungi). Each community was mixed with 1.0 × 10^5^ human embryonic kidney (HEK-293T) cells (BeNa Culture Collection Co., Ltd., China) as a standardized host background. Within each community, microorganisms were adjusted to the same concentration level (1.0 × 10^5^ copies/mL), while relative input proportions varied across species.

### Strain preparation

All strains were stored at −80°C in glycerol and activated in brain heart infusion (BHI) liquid medium (Hopebio, China) before each experiment. Strain concentrations were determined by serial dilution and plate counting. Freshly cultured HEK-293T cells were quantified using Countstar (ALIT Life Sciences, China) and used as the host matrix for mock construction.

### NGS workflows

Thawed samples were liquefied with a mucus-dissolving reagent on a vortexer at 42°C for 10 min, followed by centrifugation at 14,000 × *g*. The supernatant was discarded, and the pellet was resuspended in phosphate-buffered saline. Samples were then bead-beaten, and DNA was extracted using the Microsample Genomic DNA Extraction Kit (TIANGEN) according to the manufacturer’s instructions. DNA concentration was measured using a Qubit 4.0 fluorometer (Thermo Fisher).

For Illumina sequencing, libraries were prepared using the VAHTS Universal Plus DNA Library Prep Kit for Illumina (Vazyme, China) and sequenced as single-end sequencing (75 bp) on the Illumina NextSeq 550 platform. For MGI platform, libraries were prepared using the VAHTS Universal Plus DNA Library Prep Kit for MGI (Vazyme) and sequenced on the MGISEQ-200 platform (single-end 50 bp).

### TGS workflows

Long-read TGS was performed on the ONT platform using two workflows: ONT without host depletion and host-depleted ONT (HD-ONT). For HD-ONT, samples were treated with Heat-Labile Salt-Active Nuclease and saponin, followed by centrifugation at 17,000 × *g* for 10 min. The pellet was resuspended in lysis buffer and bead-beated using an MP FastPrep-24 5G instrument (40 s, 6 m/s). DNA was extracted using the same kit as for NGS platform. Libraries were constructed with the Rapid PCR Barcoding Kit (SQK-RPB004, Oxford Nanopore Technologies) and sequenced on GridION X5 instruments using R9.4.1 flow cells.

### Bioinformatic processing and taxonomic assignment

All sequencing data were processed using a harmonized analytic framework with platform-appropriate tools. Briefly, demultiplexing/basecalling was performed using bcl2fastq for Illumina, splitBarcode for MGI, and Guppy for ONT. For ONT reads, quality filtering was applied using a minimum Q-score threshold (>7) and a minimum read length (>200 bp). For short-read data, low-complexity reads were removed using DUST filtering, and reads were filtered by length and quality after adapter trimming.

Host reads were removed by aligning cleaned reads to the human reference genome (GRCh38) together with a plasmid sequence collection. Short reads were aligned using Bowtie2, whereas long reads were aligned using minimap2; reads mapping to the host reference were excluded. The remaining non-host reads were aligned to a curated microbial genome reference database using BWA-MEM (short reads) or minimap2 (long reads). Taxonomic classification was performed using a Lowest Common Ancestor strategy based on the NCBI taxonomy (16, 17).

### Reference database curation

Reference genomes were retrieved from NCBI RefSeq and GenBank and curated to reduce low-quality or contaminated assemblies. Assemblies were prioritized by completeness (Complete Genome > Chromosome > Scaffold > Contig) and standard quality metrics (e.g., higher N50, lower L50, fewer contigs). The final database comprised 20,484 reference genomes/sequences in total, including 5,742 bacteria, 600 fungi, 13,786 viruses, 170 archaea, and 186 parasites/protozoa.

### Read normalization, reporting threshold, and contamination control

Organism-specific results are reported as normalized read counts mapped to each species. For short-read workflows, read counts were normalized to RP20M (reads per 20 million total effective reads), whereas for the HD-ONT workflow, read counts were normalized to RP10K (reads per 10,000 total effective reads). A species was considered positive when the normalized read count exceeded the predefined platform-specific threshold, namely RP20M ≥ 3 for short-read workflows and RP10K ≥ 1 for HD-ONT. Negative and blank controls were processed in parallel for contamination monitoring. Organisms were flagged as potential contaminants if their normalized read counts exceeded the 95th percentile of the long-term background distribution derived from historical negative controls. Suspected cross-contamination was further assessed by reanalysis and manual inspection.

### Clinical performance evaluation and reference standards

Residual BALF specimens with traceable culture results were selected and stored at −80°C prior to DNA extraction. For clinical specimens, results obtained from the MGI and HD-ONT workflows were evaluated against three comparators:

1. Routine culture, which included only microorganisms that can be cultured.
2. Clinical microbiological tests (CMT), integrating results from culture and additional verification.
3. Composite reference standard (CRS), which incorporated organisms that lacked targeted testing results but were consistently detected by both sequencing workflows (MGI and HD-ONT) under the predefined reporting criteria.

In cases where discrepancies occurred among NGS, HD-ONT, and culture results, additional verification was performed when sufficient residual BALF volume was available. Discrepancies involving bacteria, fungi, Mycoplasma, Chlamydia, or non-tuberculous mycobacteria were verified by quantitative polymerase chain reaction (qPCR) using published primer sets specific to the target organisms. For Mycobacterium tuberculosis complex (MTBC), confirmation was conducted using the Xpert MTB/RIF assay performed on the GeneXpert system (Cepheid, USA). These targeted assays were used as part of the CMT comparator framework for confirmatory evaluation rather than to serve as an error-free gold standard.

Agreement between each sequencing workflow and each comparator was assessed at the organism level. Organism-level outcomes were categorized as concordant positive, sequencing positive/comparator negative, sequencing negative/comparator positive, or concordant negative, and positive percent agreement (PPA) and negative percent agreement (NPA) were calculated accordingly. In addition, discordant organisms were interpreted using a previously reported diagnostic scoring framework (18) to support clinical relevance grading.

### Statistical analysis

Correlation analyses for continuous variables were conducted using Spearman’s rank correlation, while comparisons of continuous variables were performed with the Kruskal–Wallis test. To evaluate the associations of sequencing platform and organism-level characteristics with read abundance in the mock-community experiments, a linear mixed-effects model was fitted using log-transformed normalized read counts as the outcome variable. Platform, log10 spiked input level, genome size, and GC content were included as fixed effects, and species was included as a random intercept to account for repeated measurements of the same organism across sequencing platforms. All statistical analyses and data visualizations were carried out in R software (version 4.3.3), utilizing packages such as tidyverse, dplyr, purrr, lme4, and ggplot2.

## RESULTS

### Turnaround time

In this study, the Illumina workflow required approximately 18–20 h from library preparation to result reporting, and the MGI workflow required about 14–19 h while maintaining stable batch processing. By contrast, the HD-ONT workflow was completed within 4–6 h, allowing pathogen identification to be delivered within a single working day.

### Impact of microbe-to-host ratio on sequencing outcomes

**Fig 2:**
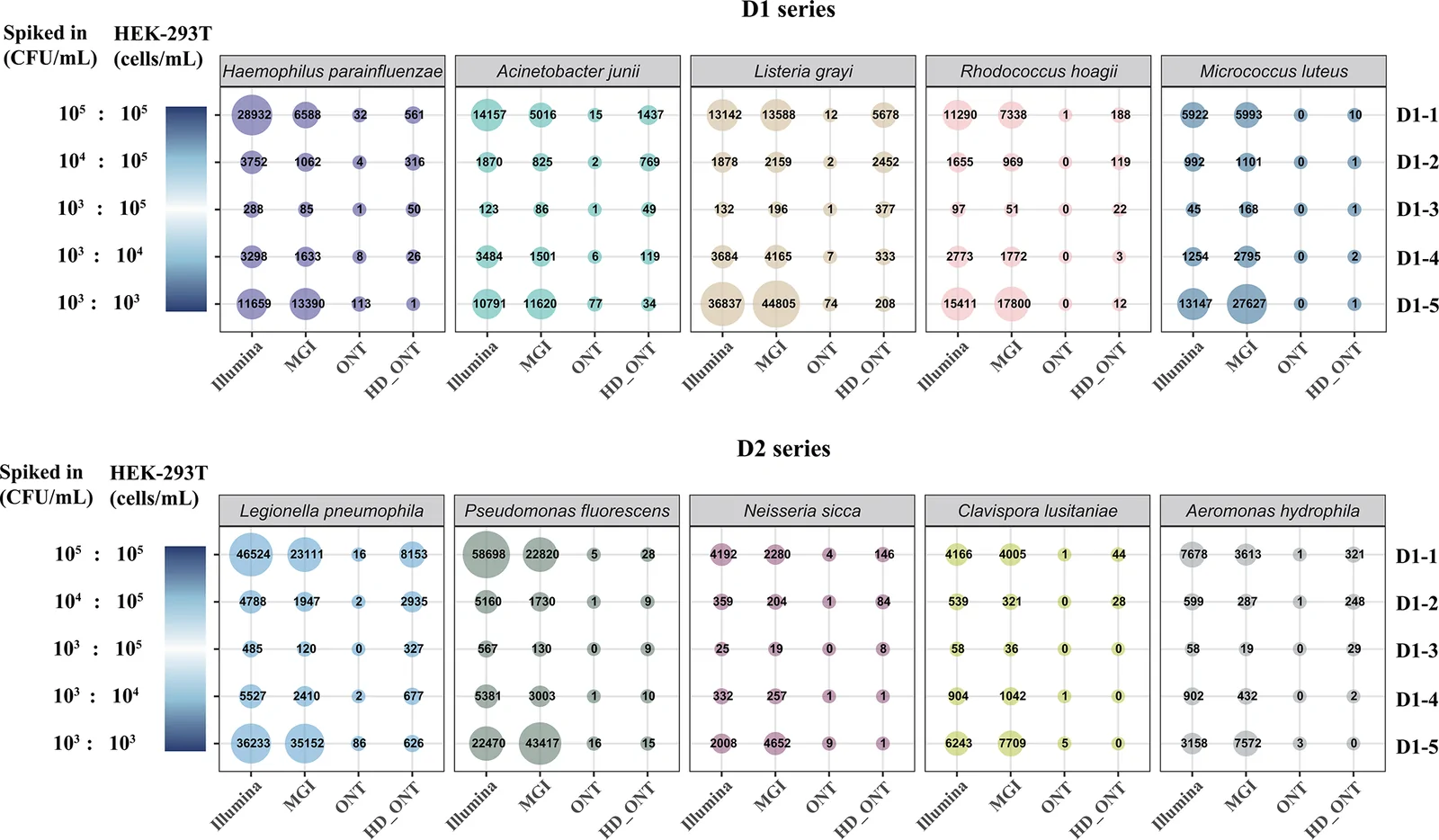
Variation in microbial read counts under different microbe-to-host DNA ratios across sequencing platforms in D1 and D2 mock communities. Bubble plots show the sequencing reads assigned to each target organism in (top) the D1 series (*Haemophilus parainfluenzae*, *Acinetobacter junii*, *Listeria grayi*, *Rhodococcus hoagii*, *Micrococcus luteus*) and (bottom) the D2 series (*Legionella pneumophila*, *Pseudomonas fluorescens*, *Neisseria sicca*, *Clavispora lusitaniae*, and *Aeromonas hydrophila*). Within each panel, the x-axis represents the sequencing platform (Illumina, MGI, ONT, and HD-ONT). The y-axis indicates the five spiking conditions (D1-1 to D1-5/D2-1 to D2-5), corresponding to the indicated combinations of bacterial/fungal concentration (CFU/mL; left column “Spiked in”) and HEK-293T cell background (cells/mL). Each circle represents the normalized read counts mapped to the labeled organism under a given condition and platform; bubble size is proportional to the organism-specific read counts, and the number inside the bubble gives the exact read count. The blue bar on the left illustrates the microbe-to-host DNA ratio, with darker blue indicating a higher microbial load relative to the HEK-293T background.

To assess the impact of microbial and host DNA abundance on sequencing outcomes, we examined gradient mixtures of microorganisms and HEK-293T cells across the D1 and D2 mock series. For both NGS (Illumina and MGI) and TGS (ONT) platforms without host depletion, the total number of microbial reads decreased progressively as the microbe-to-host ratio declined (from 10^5^:10^5^ to 10^3^:10^5^). When the microbial load remained constant at a low level but the relative proportion of host DNA was reduced, normalized read counts increased correspondingly (Fig. 2), indicating that sequencing yield was strongly dependent on the microbial-to-host DNA ratio.

The short-read platforms consistently achieved 100% detection sensitivity (50/50), accurately identifying all target microorganisms even at low input levels. Conversely, the ONT platform exhibited a false-negative rate of 43.3% (13/30) under host-dominant conditions (D1-2, D1-3, D1-4, D2-2, D2-3, D2-4), and positive detections yielded fewer than 10 reads. Incorporation of a host depletion step (HD-ONT) significantly improved performance, increasing read counts across all species and reducing the false-negative rate to 6.7% (2/30), though occasional detections were still missed for low-abundance species such as *Clavispora lusitaniae* and *Micrococcus luteus* (Table S1). These findings underscore the necessity of host DNA depletion in optimizing ONT-based metagenomic workflows.

### Capabilities of different sequencing platforms for complex community characterization

**Fig 3:**
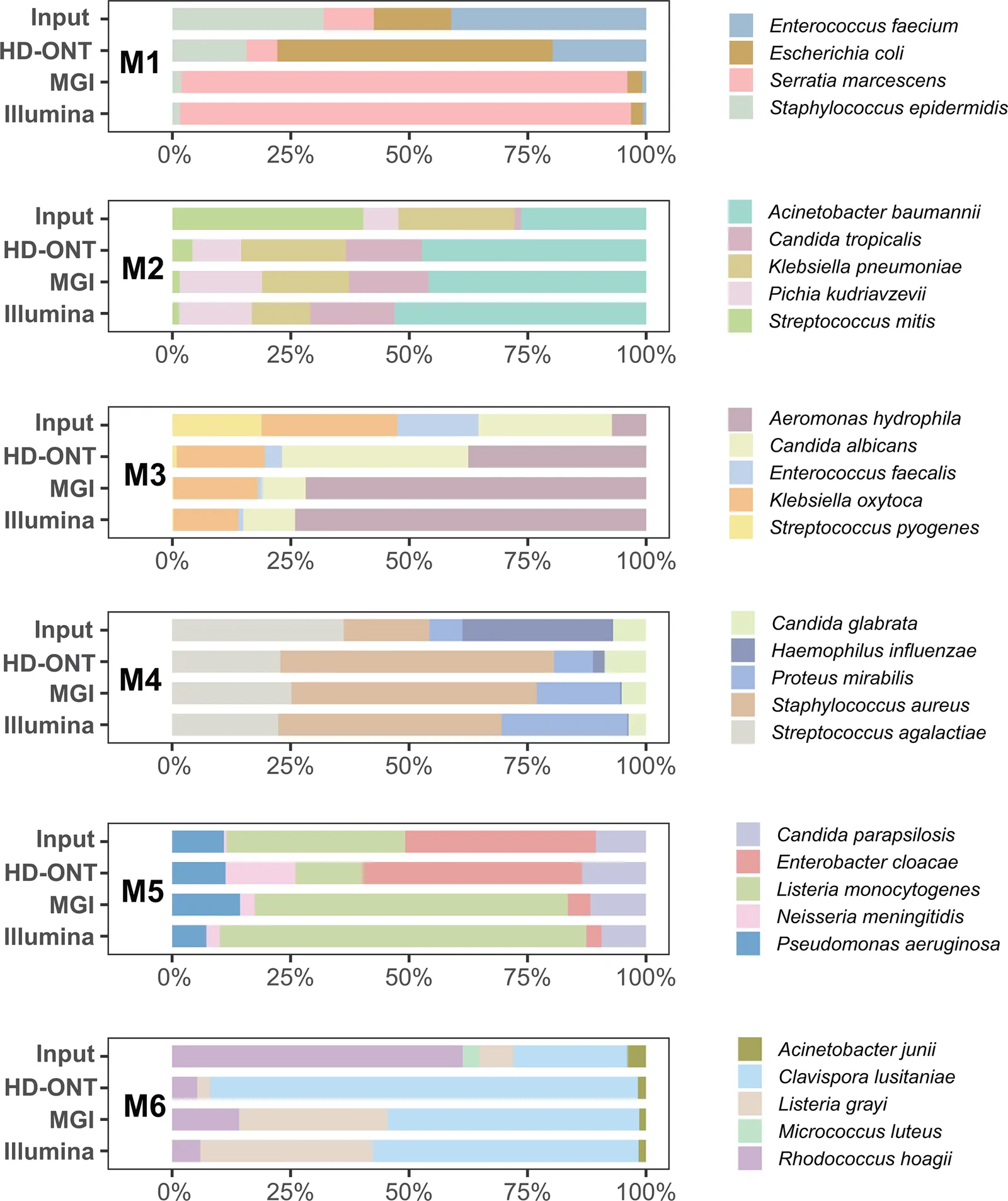
Input microbial composition and platform-specific relative read proportions for individual species in M1–M6 mock communities. Stacked horizontal bar charts show the known input composition and the read-based composition of six mock communities (M1–M6) profiled by different sequencing platforms. For each community, the bar labeled “Input” represents the spiked-in proportion of each organism in the reference mixture, whereas the bars labeled Illumina, MGI, and HD-ONT (host-depleted nanopore sequencing) represent the proportion of sequencing reads mapped to each organism. Within each bar, colored segments correspond to individual species, with colors matching the taxon labels in the legend to the right of each panel.

Six mock panels (M1–M6) were analyzed to evaluate the capacity of Illumina, MGI, and HD-ONT platforms to detect microorganisms in polymicrobial settings. Platform-level sequencing output metrics, including total reads and host/non-host read proportions for the M-series panels, are summarized in Table S3. All three platforms successfully detected all microorganisms across the panels (100% sensitivity) (Table S2), suggesting that both NGS and TGS workflows are capable of detecting multiple microorganisms in these defined benchmarking panels. However, the relative read proportions for individual species were inconsistent with their input abundance (Input) (Fig. 3), indicating that read yield did not scale linearly with the actual microbial load.

Correlation analysis further confirmed that the normalized read counts were not significantly correlated with input microbial abundance (Input) on any platform (Table S4). Moreover, only weak or inconsistent associations were observed between normalized read counts and basic microbial features such as genome size, GC content, or microbial type. To further evaluate the associations of sequencing platform and organism-level characteristics with sequencing yield, a linear mixed-effects model was fitted using log-transformed read counts as the outcome. In this model, the sequencing platform was significantly associated with read abundance, whereas input level and GC content were not significantly associated with the outcome; genome size showed a positive but non-significant trend (Table 1). These findings indicate that sequencing read counts are influenced by multiple factors and should not be directly interpreted as a quantitative proxy for pathogen abundance in multi-species samples. Notably, the two short-read platforms demonstrated highly consistent profiles (Fig. 3; Table S2), reflecting strong inter-platform reproducibility in qualitative pathogen detection.

**TABLE 1:** Linear mixed-effects model evaluating associations of sequencing platform and organism-level characteristics with log-transformed read counts in M mock-community experiments

Variable	β coefficient	SE	95% CI	*P* value
Intercept	3.764	3.926	−4.069 to 11.598	0.334
Platform (Illumina vs HD-ONT)	4.575	0.222	4.132 to 5.019	<0.001
Platform (MGI vs HD-ONT)	4.73	0.222	4.287 to 5.174	<0.001
Log10 spiked input level	0.437	0.665	−0.891 to 1.765	0.507
Genome size (Mb)	0.136	0.076	−0.016 to 0.289	0.078
GC content (%)	−0.005	0.026	−0.057 to 0.048	0.855

### Diagnostic performance for clinical samples

The diagnostic performance of NGS (MGI platform) and TGS (HD-ONT) was evaluated using 62 BALF samples, with results compared against culture, CMT, and CRS (Table 2; Table S5). When culture was used as the comparator, both sequencing platforms detected all culture-positive microorganisms except for one case of *Stenotrophomonas maltophilia*, yielding a PPA of 95.2%. However, the NPA was relatively low (44.6% for NGS, 46.4% for TGS), primarily due to the identification of additional microorganisms not recovered by culture.

**TABLE 2:** Diagnostic performance of NGS and TGS platforms based on different reference standards^*a*^

	Culture			Clinical microbiological tests (CMT)		Composite reference standard (CRS)	
NGS		＋	−	＋	−	＋	−
	＋	20	31	39	13	70	13
	−	1	25	5	39	5	45
		PPA	95.2%	PPA	88.6%	PPA	93.3%
		NPA	44.6%	NPA	75.0%	NPA	77.6%
TGS		＋	−	＋	−	＋	−
	＋	20	30	37	10	68	10
	−	1	26	7	42	7	50
		PPA	95.2%	PPA	84.1%	PPA	90.7%
		NPA	46.4%	NPA	79.2%	NPA	83.3%

^*a*^ NGS, next-generation sequencing; TGS, third-generation sequencing; PPA, positive percent agreement; NPA, negative percent agreement; +, Positive; −, Negative.

For samples with sufficient residual volume, confirmatory qPCR or GeneXpert testing provided additional support for 19 NGS-positive and 17 TGS-positive findings for the targeted organisms tested. Consequently, when assessed against CMT, the NPA increased to 75.0% for NGS and 79.2% for TGS. Additional discordant results were observed, with NGS being negative for 1 *Staphylococcus aureus*, 1 MTBC, 1 *Aspergillus niger*, and 1 *Aspergillus fumigatus*; and TGS being negative for 2 *Escherichia coli*, 1 *Klebsiella pneumoniae*, 2 *Pneumocystis jirovecii*, and 1 *Candida krusei* among organisms reported positive by CMT. The PPA for NGS (88.6%) remained slightly higher than that of TGS (84.1%).

After incorporating 31 microorganisms concordantly detected by both sequencing platforms into the CRS, PPA further increased to 93.3% for NGS and 90.7% for TGS, with NPAs of 77.6% and 83.3%, respectively. Both sequencing platforms showed superior capability in detecting samples with multiple microorganisms. Among 22 CRS-defined multi-microbial samples, culture correctly identified all microorganisms in only 2 cases, missed at least one organism in 11 cases, and was completely negative in 9 cases. In comparison, NGS detected all microorganisms in 18 of the 22 samples, missing only a single organism in the remaining 4 cases, while TGS detected all microorganisms in 17 samples and missed one organism in 5 cases (Table S5).

Overall, both NGS and TGS exhibited markedly higher PPA than culture, with NGS showing a slight advantage in PPA over TGS. Consistent trends were observed when bacteria and fungi were analyzed separately. For bacterial detection, NGS and TGS showed comparable PPA, but TGS exhibited higher NPA (Table S6). For fungal detection, NGS demonstrated higher PPA, while both platforms had similar NPA (Table S7).

## DISCUSSION

This study systematically evaluated the diagnostic performance of short-read metagenomic sequencing and long-read nanopore sequencing with host depletion (HD-ONT) in pathogen detection. Using defined mock communities and clinical BALF samples, we evaluated the effects of microbial abundance, host DNA background, and the coexistence of multiple pathogens on sequencing outcomes, and compared the clinical performance of the different platforms. Together, these analyses provide a practical comparison of NGS and TGS workflows and reveal key technical factors influencing the performance of clinical microbiological detection.

When evaluated against the CRS, both next- and third-generation sequencing approaches achieved high PPA and outperformed culture in detecting multiple pathogens. These findings align with the growing clinical consensus that metagenomic sequencing can substantially enhance pathogen detection in culture-negative or polymicrobial infection scenarios (19, 20). Traditional culture methods often fail to isolate certain pathogens due to slow growth, special nutritional requirements, or antibiotic suppression, resulting in an incomplete diagnostic spectrum. In contrast, unbiased metagenomic sequencing enables the simultaneous detection of bacteria, fungi, viruses, and atypical organisms without prior assumptions, thereby providing more comprehensive etiological information about infections (4, 21). In samples with multiple coexisting pathogens, both NGS and HD-ONT platforms achieved significantly higher detection completeness than culture, a benefit that was particularly evident in cases of polymicrobial pneumonia, ventilator-associated infections, and immunocompromised hosts (22–25). Several large-scale prospective studies have also reported that metagenomic sequencing improves diagnostic positivity rates by 50%–60% compared to conventional methods, especially for pathogens often missed by routine testing, such as *Pneumocystis jirovecii*, *Nocardia*, and obligate anaerobic bacteria (26–29).

Gradient mixture experiments demonstrated that as the proportion of host DNA increased, the number of microbial reads decreased significantly, indicating that human DNA can mask pathogen signals (8, 30), especially in cases of low-pathogen-load infections. Given that human DNA typically predominates in clinical specimens, enhancing the sensitivity of metagenomic sequencing would, in theory, require deeper sequencing. However, for long-read platforms like ONT, the lower throughput and higher per-base costs make simply increasing sequencing depth impractical. To address this, workflows often incorporate strategies to reduce the human DNA background, such as host DNA depletion for cell-rich specimens with high human DNA content (e.g., respiratory or joint fluids) and enrichment of microbial cell-free DNA (cfDNA) for low-cellularity samples (e.g., plasma and cerebrospinal fluid) (3, 31). In this study, host depletion substantially increased microbial signal detection, in line with previous reports that efficient removal of host DNA improves the signal-to-noise ratio and the diagnostic yield of clinical metagenomic sequencing (31, 32).

Despite improvements in HD-ONT performance, its sensitivity remains lower than that of NGS. Possible reasons are that, although the longer reads of HD-ONT aid in distinguishing repetitive sequences and identifying structural variants, its higher single-base error rate and homopolymer-related biases may still lead to unstable detection of some low-abundance pathogens (15); additionally, surfactants or enzymes used in the host removal process may disrupt the structure of certain pathogens (33), resulting in premature release of intracellular DNA and its unintended removal during subsequent DNase treatment. Recent comparative studies have confirmed that in metagenomic pathogen detection, Illumina platforms maintain a clear advantage in sequencing accuracy, whereas the higher per-base error rate of nanopore technology may limit species-level resolution without appropriate calibration, despite its notable advantages in speed and versatility for pathogen identification (15, 34). The turnaround time of HD-ONT is shorter than 6 h, enabling near-real-time pathogen identification and result feedback, which is particularly valuable for patients requiring rapid treatment decisions (35, 36), such as those with severe pneumonia or sepsis. Its significant turnaround time advantage makes it highly valuable in time-sensitive clinical settings, although it is not yet ready to replace short-read NGS as a routine diagnostic tool.

Our data further indicate that when multiple microorganisms coexist within a sample, the relationship between sequencing normalized read counts—whether generated by NGS or TGS—and the relative abundance of individual organisms is non-linear. Sequencing yield is shaped by a combination of biological factors (e.g., differences in cell wall composition such as thick peptidoglycan in Gram-positive bacteria or chitin-rich fungal cell walls) and technical factors (e.g., DNA extraction efficiency, library preparation chemistry, amplification bias, and platform-specific error profiles), as well as details of the bioinformatics pipeline (29, 37–40). This complexity may explain the weak correlations observed between read counts and microbial genomic features in single-factor analyses. These findings underscore a practical limitation of metagenomic sequencing: while read counts offer a general indication of organism presence and signal intensity, they fail to provide a strict quantitative measure of microbial burden. Even so, prior studies have shown that metagenomic data can still offer semi-quantitative clues—such as relative abundance patterns or the prominence of specific taxa—which, when interpreted together with clinical information and host-response markers, may help distinguish colonization from true infection (41, 42). Thus, interpretation of read-based abundance signals requires caution and should be supported by standardized laboratory workflows, inclusion of negative and positive controls, and appropriate statistical normalization to mitigate process- and platform-related biases.

Several limitations of this study should be noted. First, the clinical sample size was relatively small and primarily derived from lower respiratory tract infections, which may limit the generalizability of our findings to other infection types or sample matrices. Second, while a composite reference standard was applied to reduce the inherent bias of culture-based comparisons, potential classification uncertainties may still exist for mixed infections or low-level pathogens. Third, this study lacked a standardized virological reference standard. The number of virologically confirmed infections was limited, and the sample-processing workflow was primarily optimized for DNA pathogens, which precluded an evaluation of RNA virus detection. Therefore, we restricted the formal performance analyses (PPA/NPA) to bacterial and fungal pathogens. Finally, we did not conduct a formal cost-effectiveness analysis or evaluate broader implementation factors such as workflow integration and resource requirements, which are critical for real-world adoption. Because this study evaluated two short-read platforms and one long-read platform, the findings should be interpreted as workflow-specific rather than universally generalizable to all NGS or TGS instruments, chemistries, or bioinformatic implementations.

### Conclusion

From a clinical perspective, NGS currently provides robust and comprehensive coverage for the detection of common bacterial and fungal pathogens, offering a balance between sensitivity, throughput, and scalability. HD-ONT demonstrates potential advantages in rapid turnaround and complementary clinical application. With continued improvements in library preparation, error correction, and analytical algorithms, ONT-based workflows may evolve into a more competitive alternative for routine pathogen detection in the near future.

## Data Availability

Raw sequence data reported in this study were deposited in the Genome Sequence Archive in the National Genomics Data Center, China National Center for Bioinformation/Beijing Institute of Genomics, Chinese Academy of Sciences (GSA: PRJCA011513 and PRJCA059899), publicly accessible at https://ngdc.cncb.ac.cn/.

## SUPPLEMENTAL MATERIAL

Supplemental tables spectrum.03993-25-s0001.docx Tables S1 to S4, S6 and S7.

Table S5 spectrum.03993-25-s0002.xlsx Pathogen detection in BALF samples using different diagnostic methods.

ASM does not own the copyrights to Supplemental Material that may be linked to, or accessed through, an article. The authors have granted ASM a non-exclusive, world-wide license to publish the Supplemental Material files. Please contact the corresponding author directly for reuse.

## References

[1] Chiu CY, Miller SA. 2019. Clinical metagenomics. Nat Rev Genet 20:341–355. doi:10.1038/s41576-019-0113-7 30918369 PMC6858796

[2] Wilson MR, Sample HA, Zorn KC, Arevalo S, Yu G, Neuhaus J, Federman S, Stryke D, Briggs B, Langelier C, et al.. 2019. Clinical metagenomic sequencing for diagnosis of meningitis and encephalitis. N Engl J Med 380:2327–2340. doi:10.1056/NEJMoa1803396 31189036 PMC6764751

[3] Gu W, Deng X, Lee M, Sucu YD, Arevalo S, Stryke D, Federman S, Gopez A, Reyes K, Zorn K, Sample H, Yu G, Ishpuniani G, Briggs B, Chow ED, Berger A, Wilson MR, Wang C, Hsu E, Miller S, DeRisi JL, Chiu CY. 2021. Rapid pathogen detection by metagenomic next-generation sequencing of infected body fluids. Nat Med 27:115–124. doi:10.1038/s41591-020-1105-z 33169017 PMC9020267

[4] Miller S, Chiu C. 2021. The role of metagenomics and next-generation sequencing in infectious disease diagnosis. Clin Chem 68:115–124. doi:10.1093/clinchem/hvab173 34969106

[5] Ko KKK, Chng KR, Nagarajan N. 2022. Metagenomics-enabled microbial surveillance. Nat Microbiol 7:486–496. doi:10.1038/s41564-022-01089-w 35365786

[6] Zhao Y, Zhang W, Zhang X. 2024. Application of metagenomic next-generation sequencing in the diagnosis of infectious diseases. Front Cell Infect Microbiol 14:1458316. doi:10.3389/fcimb.2024.1458316 39619659 PMC11604630

[7] Jeon SA, Park JL, Park S-J, Kim JH, Goh S-H, Han J-Y, Kim S-Y. 2021. Comparison between MGI and Illumina sequencing platforms for whole genome sequencing. Genes Genom 43:713–724. doi:10.1007/s13258-021-01096-x 33864614

[8] Shi Y, Wang G, Lau H-H, Yu J. 2022. Metagenomic sequencing for microbial DNA in human samples: emerging technological advances. Int J Mol Sci 23:2181. doi:10.3390/ijms23042181 35216302 PMC8877284

[9] Treangen TJ, Salzberg SL. 2012. Repetitive DNA and next-generation sequencing: computational challenges and solutions. Nat Rev Genet 13:36–46. doi:10.1038/nrg3117 PMC3324860 22124482

[10] Hoang MTV, Irinyi L, Hu Y, Schwessinger B, Meyer W. 2021. Long-reads-based metagenomics in clinical diagnosis with a special focus on fungal infections. Front Microbiol 12:708550. doi:10.3389/fmicb.2021.708550 35069461 PMC8770865

[11] Oehler JB, Burns K, Warner J, Schmitz U. 2025. Long-read sequencing for the rapid response to infectious diseases outbreaks. Curr Clin Microbiol Rep 12:10. doi:10.1007/s40588-025-00247-y 40384881 PMC12081579

[12] Oehler JB, Wright H, Stark Z, Mallett AJ, Schmitz U. 2023. The application of long-read sequencing in clinical settings. Hum Genomics 17:73. doi:10.1186/s40246-023-00522-3 37553611 PMC10410870

[13] Sheka D, Alabi N, Gordon PMK. 2021. Oxford nanopore sequencing in clinical microbiology and infection diagnostics. Brief Bioinform 22:bbaa403. doi:10.1093/bib/bbaa403 33483726

[14] Wang Y, Zhao Y, Bollas A, Wang Y, Au KF. 2021. Nanopore sequencing technology, bioinformatics and applications. Nat Biotechnol 39:1348–1365. doi:10.1038/s41587-021-01108-x 34750572 PMC8988251

[15] Bejaoui S, Nielsen SH, Rasmussen A, Coia JE, Andersen DT, Pedersen TB, Møller MV, Kusk Nielsen MT, Frees D, Persson S. 2025. Comparison of Illumina and Oxford Nanopore sequencing data quality for *Clostridioides difficile* genome analysis and their application for epidemiological surveillance. BMC Genomics 26:92. doi:10.1186/s12864-025-11267-9 39885402 PMC11783910

[16] Huson DH, Auch AF, Qi J, Schuster SC. 2007. MEGAN analysis of metagenomic data. Genome Res 17:377–386. doi:10.1101/gr.5969107 17255551 PMC1800929

[17] Huson DH, Albrecht B, Bağcı C, Bessarab I, Górska A, Jolic D, Williams RBH. 2018. MEGAN-LR: new algorithms allow accurate binning and easy interactive exploration of metagenomic long reads and contigs. Biol Direct 13:6. doi:10.1186/s13062-018-0208-7 29678199 PMC5910613

[18] Miller S, Naccache SN, Samayoa E, Messacar K, Arevalo S, Federman S, Stryke D, Pham E, Fung B, Bolosky WJ, Ingebrigtsen D, Lorizio W, Paff SM, Leake JA, Pesano R, DeBiasi R, Dominguez S, Chiu CY. 2019. Laboratory validation of a clinical metagenomic sequencing assay for pathogen detection in cerebrospinal fluid. Genome Res 29:831–842. doi:10.1101/gr.238170.118 30992304 PMC6499319

[19] Li N, Cai Q, Miao Q, Song Z, Fang Y, Hu B. 2021. High-throughput metagenomics for identification of pathogens in the clinical settings. Small Methods 5:2000792. doi:10.1002/smtd.202000792 33614906 PMC7883231

[20] Gu W, Miller S, Chiu CY. 2019. Clinical metagenomic next-generation sequencing for pathogen detection. Annu Rev Pathol Mech Dis 14:319–338. doi:10.1146/annurev-pathmechdis-012418-012751 PMC6345613 30355154

[21] Wang Y, Chen T, Zhang S, Zhang L, Li Q, Lv Q, Kong D, Jiang H, Ren Y, Jiang Y, Li Y, Huang W, Liu P. 2023. Clinical evaluation of metagenomic next-generation sequencing in unbiased pathogen diagnosis of urinary tract infection. J Transl Med 21:762. doi:10.1186/s12967-023-04562-0 37891586 PMC10612365

[22] Peng J-M, Du B, Qin H-Y, Wang Q, Shi Y. 2021. Metagenomic next-generation sequencing for the diagnosis of suspected pneumonia in immunocompromised patients. J Infect 82:22–27. doi:10.1016/j.jinf.2021.01.029 33609588

[23] Xie F, Duan Z, Zeng W, Xie S, Xie M, Fu H, Ye Q, Xu T, Xie L. 2021. Clinical metagenomics assessments improve diagnosis and outcomes in community-acquired pneumonia. BMC Infect Dis 21:352. doi:10.1186/s12879-021-06039-1 33858378 PMC8047593

[24] Wu N, Ranjan P, Tao C, Liu C, Yang E, He B, Erb-Downward JR, Bo S, Zheng J, Guo C, Liu B, Sun L, Yan W, Wang M, Wang W, Wen J, Yang P, Yang L, Tian Q, Dickson RP, Shen N. 2021. Rapid identification of pathogens associated with ventilator-associated pneumonia by Nanopore sequencing. Respir Res 22:310. doi:10.1186/s12931-021-01909-3 34893078 PMC8665642

[25] Fang X, Mei Q, Fan X, Zhu C, Yang T, Zhang L, Geng S, Pan A. 2020. Diagnostic value of metagenomic next-generation sequencing for the detection of pathogens in bronchoalveolar lavage fluid in ventilator-associated pneumonia patients. Front Microbiol 11:599756. doi:10.3389/fmicb.2020.599756 33335520 PMC7736608

[26] Wu X, Li Y, Zhang M, Li M, Zhang R, Lu X, Gao W, Li Q, Xia Y, Pan P, Li Q. 2020. Etiology of severe community-acquired pneumonia in adults based on metagenomic next-generation sequencing: a prospective multicenter study. Infect Dis Ther 9:1003–1015. doi:10.1007/s40121-020-00353-y 33170499 PMC7652912

[27] Zuo Y-H, Wu Y-X, Hu W-P, Chen Y, Li Y-P, Song Z-J, Luo Z, Ju M-J, Shi M-H, Xu S-Y, Zhou H, Li X, Jie Z-J, Liu X-D, Zhang J. 2023. The clinical impact of metagenomic next-generation sequencing (mNGS) test in hospitalized patients with suspected sepsis: a multicenter prospective study. Diagnostics (Basel) 13:323. doi:10.3390/diagnostics13020323 36673134 PMC9857658

[28] Nielsen ME, Søgaard KK, Karst SM, Krarup AL, Albertsen M, Nielsen HL. 2025. Application of rapid Nanopore metagenomic cell-free DNA sequencing to diagnose bloodstream infections: a prospective observational study. Microbiol Spectr 13:e0329524. doi:10.1128/spectrum.03295-24 40135889 PMC12054037

[29] Boers SA, Jansen R, Hays JP. 2019. Understanding and overcoming the pitfalls and biases of next-generation sequencing (NGS) methods for use in the routine clinical microbiological diagnostic laboratory. Eur J Clin Microbiol Infect Dis 38:1059–1070. doi:10.1007/s10096-019-03520-3 30834996 PMC6520317

[30] Pereira-Marques J, Hout A, Ferreira RM, Weber M, Pinto-Ribeiro I, van Doorn L-J, Knetsch CW, Figueiredo C. 2019. Impact of host DNA and sequencing depth on the taxonomic resolution of whole metagenome sequencing for microbiome analysis. Front Microbiol 10:1277. doi:10.3389/fmicb.2019.01277 31244801 PMC6581681

[31] Gan M, Wu B, Yan G, Li G, Sun L, Lu G, Zhou W. 2021. Combined nanopore adaptive sequencing and enzyme-based host depletion efficiently enriched microbial sequences and identified missing respiratory pathogens. BMC Genomics 22:732. doi:10.1186/s12864-021-08023-0 34627155 PMC8501638

[32] Charalampous T, Kay GL, Richardson H, Aydin A, Baldan R, Jeanes C, Rae D, Grundy S, Turner DJ, Wain J, Leggett RM, Livermore DM, O’Grady J. 2019. Nanopore metagenomics enables rapid clinical diagnosis of bacterial lower respiratory infection. Nat Biotechnol 37:783–792. doi:10.1038/s41587-019-0156-5 31235920

[33] Longhi G, Argentini C, Fontana F, Tarracchini C, Mancabelli L, Lugli GA, Alessandri G, Lahner E, Pivetta G, Turroni F, Ventura M, Milani C. 2024. Saponin treatment for eukaryotic DNA depletion alters the microbial DNA profiles by reducing the abundance of Gram-negative bacteria in metagenomics analyses. Microbiome Res Rep 3:4. doi:10.20517/mrr.2023.02 38455080 PMC10917613

[34] Lorenzin G, Carlin M. 2025. Comparative meta-analysis of long-read and short-read sequencing for metagenomic profiling of the lower respiratory tract infections. Microorganisms 13:2366. doi:10.3390/microorganisms13102366 41156825 PMC12566492

[35] Chen J, Xu F. 2023. Application of nanopore sequencing in the diagnosis and treatment of pulmonary infections. Mol Diagn Ther 27:685–701. doi:10.1007/s40291-023-00669-8 37563539 PMC10590290

[36] Harris PNA, Bauer MJ, Lüftinger L, Beisken S, Forde BM, Balch R, Cotta M, Schlapbach L, Raman S, Shekar K, Kruger P, Lipman J, Bialasiewicz S, Coin L, Roberts JA, Paterson DL, Irwin AD. 2024. Rapid nanopore sequencing and predictive susceptibility testing of positive blood cultures from intensive care patients with sepsis. Microbiol Spectr 12:e03065-23. doi:10.1128/spectrum.03065-23 38193658 PMC10846127

[37] Browne PD, Nielsen TK, Kot W, Aggerholm A, Gilbert MTP, Puetz L, Rasmussen M, Zervas A, Hansen LH. 2020. GC bias affects genomic and metagenomic reconstructions, underrepresenting GC-poor organisms. Gigascience 9:giaa008. doi:10.1093/gigascience/giaa008 32052832 PMC7016772

[38] van Dijk EL, Jaszczyszyn Y, Thermes C. 2014. Library preparation methods for next-generation sequencing: tone down the bias. Exp Cell Res 322:12–20. doi:10.1016/j.yexcr.2014.01.008 24440557

[39] SoRelle JA, Wachsmann M, Cantarel BL. 2020. Assembling and validating bioinformatic pipelines for next-generation sequencing clinical assays. Arch Pathol Lab Med 144:1118–1130. doi:10.5858/arpa.2019-0476-RA 32045276

[40] Brooks JP, Edwards DJ, Harwich MD Jr, Rivera MC, Fettweis JM, Serrano MG, Reris RA, Sheth NU, Huang B, Girerd P, Strauss JF 3rd, Jefferson KK, Buck GA, Vaginal Microbiome Consortium. 2015. The truth about metagenomics: quantifying and counteracting bias in 16S rRNA studies. BMC Microbiol 15:66. doi:10.1186/s12866-015-0351-6 25880246 PMC4433096

[41] Janes VA, Matamoros S, Munk P, Clausen P, Koekkoek SM, Koster LAM, Jakobs ME, de Wever B, Visser CE, Aarestrup FM, Lund O, de Jong MD, Bossuyt PMM, Mende DR, Schultsz C. 2022. Metagenomic DNA sequencing for semi-quantitative pathogen detection from urine: a prospective, laboratory-based, proof-of-concept study. Lancet Microbe 3:e588–e597. doi:10.1016/S2666-5247(22)00088-X 35688170

[42] Jiang Z, Gai W, Zhang X, Zheng Y, Jin X, Han Z, Ao G, He J, Shu D, Liu X, Zhou Y, Hua Z. 2024. Clinical performance of metagenomic next-generation sequencing for diagnosis of pulmonary *Aspergillus* infection and colonization. Front Cell Infect Microbiol 14:1345706. doi:10.3389/fcimb.2024.1345706 38606292 PMC11007027

