# Supplementary material for: Benchmarking next- versus third-generation sequencing in metagenomics: performance metrics and diagnostic efficacy
Source: Microbiol Spectr. 2026 Jun 3;14(7):e03993-25. doi: 10.1128/spectrum.03993-25 (PMC13339908; doi:10.1128/spectrum.03993-25)
Supplement: Supplemental tables — Tables S1 to S4, S6 and S7. [file spectrum.03993-25-s0001.docx]

Supplemental Table 1. Detection of D1 and D2 Communities on Illumina, MGI, ONT and HD-ONT Platforms

| **Reference group** | **HEK-293T cells/mL** | **Spiked in CFU/mL** | **Species** | **Features** | | | | **Illumina** | | **MGI** | | **ONT** | | **HD-ONT** | |
| --- | --- | --- | --- | --- | --- | --- | --- | --- | --- | --- | --- | --- | --- | --- | --- |
|  |  |  |  |  |  |  |  | **(Without host depletion)** | | **(Without host depletion)** | | **(Without host depletion)** | | **(Host depletion)** | |
|  |  |  |  | **Genus** | **Type** | **Genome size (Mb)** | **GC%** | **RP20M** | **Result** | **RP20M** | **Result** | **RP10K** | **Result** | **RP10K** | **Result** |
| D1-1 | 1.00E+05 | 3.00E+05 | *Haemophilus parainfluenzae* | *Haemophilus* | G- | 2 | 39.5 | 28932 | TP | 6588 | TP | 32 | TP | 561 | TP |
|  |  | 2.00E+05 | *Acinetobacter junii* | *Acinetobacter* | G- | 3.4 | 38.7 | 14157 | TP | 5016 | TP | 15 | TP | 1437 | TP |
|  |  | 4.00E+05 | *Listeria grayi* | *Listeria* | G+ | 2.7 | 41.6 | 13142 | TP | 13588 | TP | 12 | TP | 5678 | TP |
|  |  | 1.50E+05 | *Rhodococcus hoagii* | *Rhodococcus* | G+ | 5.3 | 68.7 | 11290 | TP | 7338 | TP | 1 | TP | 188 | TP |
|  |  | 1.50E+04 | *Micrococcus luteus* | *Micrococcus* | G+ | 2.5 | 73 | 5922 | TP | 5993 | TP | 0 | FN | 10 | TP |
| D1-2 | 1.00E+05 | 3.00E+04 | *Haemophilus parainfluenzae* | *Haemophilus* | G- | 2 | 39.5 | 3752 | TP | 1062 | TP | 4 | TP | 316 | TP |
|  |  | 2.00E+04 | *Acinetobacter junii* | *Acinetobacter* | G- | 3.4 | 38.7 | 1870 | TP | 825 | TP | 2 | TP | 769 | TP |
|  |  | 4.00E+04 | *Listeria grayi* | *Listeria* | G+ | 2.7 | 41.6 | 1878 | TP | 2159 | TP | 2 | TP | 2452 | TP |
|  |  | 1.50E+04 | *Rhodococcus hoagii* | *Rhodococcus* | G+ | 5.3 | 68.7 | 1655 | TP | 969 | TP | 0 | FN | 119 | TP |
|  |  | 1.50E+03 | *Micrococcus luteus* | *Micrococcus* | G+ | 2.5 | 73 | 992 | TP | 1101 | TP | 0 | FN | 1 | TP |
| D1-3 | 1.00E+05 | 3.00E+03 | *Haemophilus parainfluenzae* | *Haemophilus* | G- | 2 | 39.5 | 288 | TP | 85 | TP | 1 | TP | 50 | TP |
|  |  | 2.00E+03 | *Acinetobacter junii* | *Acinetobacter* | G- | 3.4 | 38.7 | 123 | TP | 86 | TP | 1 | TP | 49 | TP |
|  |  | 4.00E+03 | *Listeria grayi* | *Listeria* | G+ | 2.7 | 41.6 | 132 | TP | 196 | TP | 1 | TP | 377 | TP |
|  |  | 1.50E+03 | *Rhodococcus hoagii* | *Rhodococcus* | G+ | 5.3 | 68.7 | 97 | TP | 51 | TP | 0 | FN | 22 | TP |
|  |  | 1.50E+02 | *Micrococcus luteus* | *Micrococcus* | G+ | 2.5 | 73 | 45 | TP | 168 | TP | 0 | FN | 1 | TP |
| D1-4 | 1.00E+04 | 3.00E+03 | *Haemophilus parainfluenzae* | *Haemophilus* | G- | 2 | 39.5 | 3298 | TP | 1633 | TP | 8 | TP | 26 | TP |
|  |  | 2.00E+03 | *Acinetobacter junii* | *Acinetobacter* | G- | 3.4 | 38.7 | 3484 | TP | 1501 | TP | 6 | TP | 119 | TP |
|  |  | 4.00E+03 | *Listeria grayi* | *Listeria* | G+ | 2.7 | 41.6 | 3684 | TP | 4165 | TP | 7 | TP | 333 | TP |
|  |  | 1.50E+03 | *Rhodococcus hoagii* | *Rhodococcus* | G+ | 5.3 | 68.7 | 2773 | TP | 1772 | TP | 0 | FN | 3 | TP |
|  |  | 1.50E+02 | *Micrococcus luteus* | *Micrococcus* | G+ | 2.5 | 73 | 1254 | TP | 2795 | TP | 0 | FN | 2 | TP |
| D1-5 | 1.00E+03 | 3.00E+03 | *Haemophilus parainfluenzae* | *Haemophilus* | G- | 2 | 39.5 | 11659 | TP | 13390 | TP | 113 | TP | 1 | TP |
|  |  | 2.00E+03 | *Acinetobacter junii* | *Acinetobacter* | G- | 3.4 | 38.7 | 10791 | TP | 11620 | TP | 77 | TP | 34 | TP |
|  |  | 4.00E+03 | *Listeria grayi* | *Listeria* | G+ | 2.7 | 41.6 | 36837 | TP | 44805 | TP | 74 | TP | 208 | TP |
|  |  | 1.50E+03 | *Rhodococcus hoagii* | *Rhodococcus* | G+ | 5.3 | 68.7 | 15411 | TP | 17800 | TP | 0 | FN | 12 | TP |
|  |  | 1.50E+02 | *Micrococcus luteus* | *Micrococcus* | G+ | 2.5 | 73 | 13147 | TP | 27627 | TP | 0 | FN | 1 | TP |
| D2-1 | 1.00E+05 | 1.00E+05 | *Legionella pneumophila* | *Legionella* | G- | 3.4 | 38.3 | 46524 | TP | 23111 | TP | 16 | TP | 8153 | TP |
|  |  | 1.50E+05 | *Pseudomonas fluorescens* | *Pseudomonas* | G- | 6.3 | 60.2 | 58698 | TP | 22820 | TP | 5 | TP | 28 | TP |
|  |  | 1.50E+05 | *Neisseria sicca* | *Neisseria* | G- | 2.5 | 51.1 | 4192 | TP | 2280 | TP | 4 | TP | 146 | TP |
|  |  | 4.00E+03 | *Clavispora lusitaniae* | *Clavispora* | Fungus | 12 | 44.5 | 4166 | TP | 4005 | TP | 1 | TP | 44 | TP |
|  |  | 1.50E+05 | *Aeromonas hydrophila* | *Aeromonas* | G- | 4.9 | 61.2 | 7678 | TP | 3613 | TP | 1 | TP | 321 | TP |
| D2-2 | 1.00E+05 | 1.00E+04 | *Legionella pneumophila* | *Legionella* | G- | 3.4 | 38.3 | 4788 | TP | 1947 | TP | 2 | TP | 2935 | TP |
|  |  | 1.50E+04 | *Pseudomonas fluorescens* | *Pseudomonas* | G- | 6.3 | 60.2 | 5160 | TP | 1730 | TP | 1 | TP | 9 | TP |
|  |  | 1.50E+04 | *Neisseria sicca* | *Neisseria* | G- | 2.5 | 51.1 | 359 | TP | 204 | TP | 1 | TP | 84 | TP |
|  |  | 4.00E+02 | *Clavispora lusitaniae* | *Clavispora* | Fungus | 12 | 44.5 | 539 | TP | 321 | TP | 0 | FN | 28 | TP |
|  |  | 1.50E+04 | *Aeromonas hydrophila* | *Aeromonas* | G- | 4.9 | 61.2 | 599 | TP | 287 | TP | 1 | TP | 248 | TP |
| D2-3 | 1.00E+05 | 1.00E+03 | *Legionella pneumophila* | *Legionella* | G- | 3.4 | 38.3 | 485 | TP | 120 | TP | 0 | FN | 327 | TP |
|  |  | 1.50E+03 | *Pseudomonas fluorescens* | *Pseudomonas* | G- | 6.3 | 60.2 | 567 | TP | 130 | TP | 0 | FN | 9 | TP |
|  |  | 1.50E+03 | *Neisseria sicca* | *Neisseria* | G- | 2.5 | 51.1 | 25 | TP | 19 | TP | 0 | FN | 8 | TP |
|  |  | 4.00E+01 | *Clavispora lusitaniae* | *Clavispora* | Fungus | 12 | 44.5 | 58 | TP | 36 | TP | 0 | FN | 0 | FN |
|  |  | 1.50E+03 | *Aeromonas hydrophila* | *Aeromonas* | G- | 4.9 | 61.2 | 58 | TP | 19 | TP | 0 | FN | 29 | TP |
| D2-4 | 1.00E+04 | 1.00E+03 | *Legionella pneumophila* | *Legionella* | G- | 3.4 | 38.3 | 5527 | TP | 2410 | TP | 2 | TP | 677 | TP |
|  |  | 1.50E+03 | *Pseudomonas fluorescens* | *Pseudomonas* | G- | 6.3 | 60.2 | 5381 | TP | 3003 | TP | 1 | TP | 10 | TP |
|  |  | 1.50E+03 | *Neisseria sicca* | *Neisseria* | G- | 2.5 | 51.1 | 332 | TP | 257 | TP | 1 | TP | 1 | TP |
|  |  | 4.00E+01 | *Clavispora lusitaniae* | *Clavispora* | Fungus | 12 | 44.5 | 904 | TP | 1042 | TP | 1 | TP | 0 | FN |
|  |  | 1.50E+03 | *Aeromonas hydrophila* | *Aeromonas* | G- | 4.9 | 61.2 | 902 | TP | 432 | TP | 0 | FN | 2 | TP |
| D2-5 | 1.00E+03 | 1.00E+03 | *Legionella pneumophila* | *Legionella* | G- | 3.4 | 38.3 | 36233 | TP | 35152 | TP | 86 | TP | 626 | TP |
|  |  | 1.50E+03 | *Pseudomonas fluorescens* | *Pseudomonas* | G- | 6.3 | 60.2 | 22470 | TP | 43417 | TP | 16 | TP | 15 | TP |
|  |  | 1.50E+03 | *Neisseria sicca* | *Neisseria* | G- | 2.5 | 51.1 | 2008 | TP | 4652 | TP | 9 | TP | 1 | TP |
|  |  | 4.00E+01 | *Clavispora lusitaniae* | *Clavispora* | Fungus | 12 | 44.5 | 6243 | TP | 7709 | TP | 5 | TP | 0 | FN |
|  |  | 1.50E+03 | *Aeromonas hydrophila* | *Aeromonas* | G- | 4.9 | 61.2 | 3158 | TP | 7572 | TP | 3 | TP | 0 | FN |

Note: TP, true positive; FP, false positive; TN, true negative; FN, false negative.

Supplemental Table 2. Detection of M1-M6 Communities on Illumina, MGI and HD-ONT Platforms

| **Reference group** | **HEK-293T cells/mL** | **Spiked in CFU/mL** | **Species** | **Features** | | | | **Illumina** | | **MGI** | | **HD-ONT** | |
| --- | --- | --- | --- | --- | --- | --- | --- | --- | --- | --- | --- | --- | --- |
|  |  |  |  | **Genus** | **Type** | **Genome size (Mb)** | **GC%** | **RP20M** | **Result** | **RP20M** | **Result** | **RP10K** | **Result** |
| M1 | 1.00E+05 | 4.30E+05 | *Enterococcus faecium* | *Enterococcus* | G+ | 2.9 | 37.8 | 30130 | TP | 33434 | TP | 1630 | TP |
|  |  | 1.11E+05 | *Serratia marcescens* | *Serratia* | G- | 5.2 | 59.7 | 4012288 | TP | 3477695 | TP | 539 | TP |
|  |  | 1.70E+05 | *Escherichia coli* | *Escherichia* | G- | 5.1 | 50.6 | 105154 | TP | 114146 | TP | 4780 | TP |
|  |  | 3.33E+05 | *Staphylococcus epidermidis* | *Staphylococcus* | G+ | 2.5 | 32 | 66030 | TP | 71221 | TP | 1285 | TP |
| M2 | 1.00E+05 | 5.70E+05 | *Acinetobacter baumannii* | *Acinetobacter* | G- | 4 | 39 | 447756 | TP | 386776 | TP | 4136 | TP |
|  |  | 5.30E+05 | *Klebsiella pneumoniae* | *Klebsiella* | G- | 5.6 | 57.1 | 104194 | TP | 154936 | TP | 1930 | TP |
|  |  | 3.00E+04 | *Candida tropicalis* | *Candida* | Fungus | 14.7 | 33.3 | 150108 | TP | 141447 | TP | 1402 | TP |
|  |  | 1.60E+05 | *Pichia kudriavzevii* | *Pichia* | Fungus | 10.9 | 38.3 | 129317 | TP | 146621 | TP | 899 | TP |
|  |  | 8.70E+05 | *Streptococcus mitis* | *Streptococcus* | G+ | 2 | 40 | 11840 | TP | 12697 | TP | 366 | TP |
| M3 | 1.00E+05 | 2.43E+05 | *Enterococcus faecalis* | *Enterococcus* | G+ | 3 | 37.4 | 8742 | TP | 9150 | TP | 319 | TP |
|  |  | 4.00E+05 | *Candida albicans* | *Candida* | Fungus | 14.7 | 33.6 | 91306 | TP | 88671 | TP | 3435 | TP |
|  |  | 4.07E+05 | *Klebsiella oxytoca* | *Klebsiella* | G- | 6 | 55 | 114522 | TP | 170890 | TP | 1624 | TP |
|  |  | 1.03E+05 | *Aeromonas hydrophila* | *Aeromonas* | G- | 4.9 | 61.2 | 618241 | TP | 689908 | TP | 3283 | TP |
|  |  | 2.67E+05 | *Streptococcus pyogenes* | *Streptococcus* | G+ | 1.8 | 38.4 | 1685 | TP | 1630 | TP | 77 | TP |
| M4 | 1.00E+05 | 1.15E+05 | *Candida glabrata* | *Candida* | Fungus | 12.6 | 38.6 | 31259 | TP | 36646 | TP | 542 | TP |
|  |  | 3.00E+05 | *Staphylococcus aureus* | *Staphylococcus* | G+ | 2.8 | 32.7 | 400087 | TP | 369075 | TP | 3583 | TP |
|  |  | 1.16E+05 | *Proteus mirabilis* | *Proteus* | G- | 4 | 38.9 | 224971 | TP | 125370 | TP | 511 | TP |
|  |  | 6.00E+05 | *Streptococcus agalactiae* | *Streptococcus* | G+ | 2.1 | 35.4 | 190012 | TP | 179500 | TP | 1417 | TP |
|  |  | 5.27E+05 | *Haemophilus influenzae* | *Haemophilus* | G- | 1.8 | 38 | 2131 | TP | 2311 | TP | 151 | TP |
| M5 | 1.00E+05 | 8.03E+05 | *Listeria monocytogenes* | *Listeria* | G+ | 3 | 37.9 | 1356601 | TP | 1068750 | TP | 1124 | TP |
|  |  | 8.57E+05 | *Enterobacter cloacae* | *Enterobacter* | G- | 5 | 55 | 56267 | TP | 77102 | TP | 3646 | TP |
|  |  | 2.33E+05 | *Pseudomonas aeruginosa* | *Pseudomonas* | G- | 6.6 | 66.2 | 127125 | TP | 232018 | TP | 889 | TP |
|  |  | 2.25E+05 | *Candida parapsilosis* | *Candida* | Fungus | 13 | 38.7 | 164490 | TP | 189696 | TP | 1063 | TP |
|  |  | 1.14E+04 | *Neisseria meningitidis* | *Neisseria* | G- | 2.1 | 51.7 | 49024 | TP | 50042 | TP | 1162 | TP |
| M6 | 1.00E+05 | 1.43E+05 | *Micrococcus luteus* | *Micrococcus* | G+ | 2.5 | 73 | 465 | TP | 1782 | TP | 1 | TP |
|  |  | 2.47E+06 | *Rhodococcus hoagii* | *Rhodococcus* | G+ | 5.3 | 68.7 | 58494 | TP | 189278 | TP | 484 | TP |
|  |  | 2.87E+05 | *Listeria grayi* | *Listeria* | G+ | 2.7 | 41.6 | 359189 | TP | 418869 | TP | 250 | TP |
|  |  | 1.57E+05 | *Acinetobacter junii* | *Acinetobacter* | G- | 3.4 | 38.7 | 15478 | TP | 19271 | TP | 157 | TP |
|  |  | 9.70E+05 | *Clavispora lusitaniae* | *Clavispora* | Fungus | 11.9 | 44.5 | 554119 | TP | 711932 | TP | 8244 | TP |

Supplemental Table 3. Platform-level sequencing output metrics of M1-M6 Communities

| Group | platform | Raw reads | Clean reads | Host reads | Nonhost reads | Nonhost rate |
| --- | --- | --- | --- | --- | --- | --- |
| M1 | HD-ONT | 140000 | 139277 | 5127 | 134150 | 0.96 |
| M1 | Illumina | 20879263 | 19489570 | 12222724 | 7266846 | 0.37 |
| M1 | MGI | 47895682 | 36517757 | 24204491 | 12313266 | 0.33 |
| M2 | HD-ONT | 172000 | 169425 | 3254 | 166171 | 0.98 |
| M2 | Illumina | 21425126 | 19707208 | 16600745 | 3106463 | 0.16 |
| M2 | MGI | 49214593 | 44064131 | 37900552 | 6163579 | 0.14 |
| M3 | HD-ONT | 212000 | 207490 | 4068 | 203422 | 0.98 |
| M3 | Illumina | 25085456 | 24098599 | 20235350 | 3863249 | 0.16 |
| M3 | MGI | 66701967 | 57523565 | 47370166 | 10153399 | 0.18 |
| M4 | HD-ONT | 208000 | 205084 | 2393 | 202691 | 0.99 |
| M4 | Illumina | 25113902 | 23414434 | 19996363 | 3418071 | 0.15 |
| M4 | MGI | 67872887 | 58340406 | 52072474 | 6267932 | 0.11 |
| M5 | HD-ONT | 224000 | 221253 | 1650 | 219603 | 0.99 |
| M5 | Illumina | 34363506 | 31297786 | 24759638 | 6538148 | 0.21 |
| M5 | MGI | 45209671 | 38800034 | 31809018 | 6991016 | 0.18 |
| M6 | HD-ONT | 264000 | 260737 | 1994 | 258743 | 0.99 |
| M6 | Illumina | 31243175 | 29910560 | 16589806 | 13320754 | 0.45 |
| M6 | MGI | 53796580 | 37090966 | 25319029 | 11771937 | 0.32 |

Supplemental Table 4. Correlation Analyses of Sequencing Read Counts with Microbial and Platform-related Characteristics Across Illumina, MGI, and HD-ONT Platforms

| Platform | Variable | Test | ρ / η² | P value |
| --- | --- | --- | --- | --- |
| HD-ONT | Illumina | Spearman | 0.49 | 6.95E-03 |
|  | MGI | Spearman | 0.45 | 0.0143 |
|  | Group(M1-M6) | Kruskal-Wallis | 0.136 | 0.579 |
|  | Microbial abundance (Input) | Spearman | 0.241 | 0.208 |
|  | Genome size | Spearman | 0.399 | 0.0322 |
|  | GC content | Spearman | -0.071 | 0.713 |
|  | Microbial type (G+,G-,Fungus) | Kruskal-Wallis | 0.114 | 2.02E-01 |
| Illumina | MGI | Spearman | 0.944 | 1.66E-14 |
|  | HD-ONT | Spearman | 0.49 | 0.00695 |
|  | Group(M1-M6) | Kruskal-Wallis | 0.036 | 0.961 |
|  | Microbial abundance (Input) | Spearman | -0.017 | 0.929 |
|  | Genome size | Spearman | 0.363 | 0.0529 |
|  | GC content | Spearman | 0.038 | 0.843 |
|  | Microbial type (G+,G-,Fungus) | Kruskal-Wallis | 0.056 | 0.459 |
| MGI | Illumina | Spearman | 0.944 | 1.66E-14 |
|  | HD-ONT | Spearman | 0.45 | 0.0143 |
|  | Group(M1-M6) | Kruskal-Wallis | 0.054 | 0.912 |
|  | Microbial abundance (Input) | Spearman | 0.119 | 0.538 |
|  | Genome size | Spearman | 0.406 | 0.0287 |
|  | GC content | Spearman | 0.216 | 0.26 |
|  | Microbial type (G+,G-,Fungus) | Kruskal-Wallis | 0.032 | 0.636 |

Supplemental Table 6. Diagnostic Performance of NGS and TGS Platforms for Bacterial Detection

| **NGS** | **Culture** | | | **Clinical microbiological tests (CMT)** | | | **Composite reference** | | |
| --- | --- | --- | --- | --- | --- | --- | --- | --- | --- |
|  |  |  |  |  |  |  | standards (CRS) | | |
|  |  | ＋ | − |  | ＋ | − |  | ＋ | − |
|  | ＋ | 14 | 26 |  | 26 | 10 |  | 47 | 10 |
|  | − | 1 | 31 |  | 3 | 40 |  | 3 | 50 |
|  |  |  |  |  |  |  |  |  |  |
|  |  | Sensitivity | 93.30% |  | PPA | 89.70% |  | PPA | 94.00% |
|  |  | Specificity | 54.40% |  | NPA | 80.00% |  | NPA | 83.30% |
| **TGS** | **Culture** | | | **Clinical microbiological tests (CMT)** | | | **Composite reference** | | |
|  |  |  |  |  |  |  | standards (CRS) | | |
|  |  | ＋ | − |  | ＋ | − |  | ＋ | − |
|  | ＋ | 14 | 24 |  | 25 | 5 |  | 46 | 5 |
|  | − | 1 | 32 |  | 4 | 40 |  | 4 | 52 |
|  |  |  |  |  |  |  |  |  |  |
|  |  | Sensitivity | 93.30% |  | PPA | 86.20% |  | PPA | 92.00% |
|  |  | Specificity | 57.10% |  | NPA | 88.90% |  | NPA | 91.20% |

Supplemental Table 7. Diagnostic Performance of NGS and TGS Platforms for Fungal Detection

| **NGS** | **Culture** | | | **Clinical microbiological tests (CMT)** | | | **Composite reference** | | |
| --- | --- | --- | --- | --- | --- | --- | --- | --- | --- |
|  |  |  |  |  |  |  | **standards (CRS)** | | |
|  |  | ＋ | − |  | ＋ | − |  | ＋ | − |
|  | ＋ | 4 | 8 |  | 11 | 4 |  | 17 | 4 |
|  | − | 0 | 48 |  | 2 | 52 |  | 2 | 55 |
|  |  |  |  |  |  |  |  |  |  |
|  |  | Sensitivity | 100% |  | PPA | 84.60% |  | PPA | 89.50% |
|  |  | Specificity | 85.70% |  | NPA | 92.90% |  | NPA | 93.20% |
| **TGS** | **Culture** | | | **Clinical microbiological tests (CMT)** | | | **Composite reference** | | |
|  |  |  |  |  |  |  | **standards (CRS)** | | |
|  |  | ＋ | − |  | ＋ | − |  | ＋ | − |
|  | ＋ | 4 | 11 |  | 10 | 5 |  | 16 | 5 |
|  | − | 0 | 48 |  | 3 | 52 |  | 3 | 55 |
|  |  |  |  |  |  |  |  |  |  |
|  |  | Sensitivity | 100% |  | PPA | 76.90% |  | PPA | 84.20% |
|  |  | Specificity | 81.40% |  | NPA | 91.20% |  | NPA | 91.70% |
